# A holobiont view on thrombosis: unravelling the microbiota's influence on arterial thrombus growth

**DOI:** 10.15698/mic2020.01.704

**Published:** 2020-01-02

**Authors:** Giulia Pontarollo, Klytaimnistra Kiouptsi, Christoph Reinhardt

**Affiliations:** 1 Center for Thrombosis and Hemostasis (CTH), University Medical Center Mainz, Johannes Gutenberg University of Mainz, Langenbeckstrasse 1, 55131 Mainz, Germany.; 2 German Center for Cardiovascular Research (DZHK), Partner Site RheinMain, Mainz, Germany.

**Keywords:** microbiota, germ-free, arterial thrombosis, atherothrombosis, carotid artery, atherosclerosis mouse models, late atherosclerosis, cholesterol, platelet

## Abstract

The commensal microbiota has co-evolved with its host, colonizing all body surfaces. Therefore, this microbial ecosystem is intertwined with host physiology at multiple levels. While it is evident that microbes that reach the blood stream can trigger thrombus formation, it remains poorly explored if the wealth of microbes that colonize the body surfaces of the mammalian host can be regarded as a modifier of cardiovascular disease (CVD) development. To experimentally address the microbiota's role in the development of atherosclerotic lesions and arterial thrombosis, we generated a germ-free (GF) low-density lipoprotein receptor-deficient (*Ldlr^−/−^*) atherosclerosis mouse model (Kiouptsi et al., mBio, 2019) and explored the role of nutritional composition on arterial thrombogenesis.

From a perspective of cultural evolution, during the past decades Western societies have faced an enormous shift from traditional fibre-rich nutrition to the consumption of industrial foods that severely affects the diversity and function of the gut microbiota. To a large extent, the mutualistic nature of this microbial ecosystem with its host relies on intact barriers that are typically are formed by the mucus layer, the integrity of epithelial cell layers and by a tightly regulated and multifaceted immune vigilance but is also determined by nutritional factors shaping the diversity of the commensal microbiota. These efficient barriers are disrupted by infectious pathogens and in case of injury. Related to infection, the concept of immunothrombosis views blood clotting as an intravascular effector pathway of innate immunity, constituting a physiologic response that prevents the invasion and dissemination of microbes but becoming detrimental to the host if exceeding, e.g. in sepsis. These microorganisms produce foreign products that, dependent on dietary habits, constitute a continuous stimulus of low-grade inflammation and at the same time possess an enormous metabolic capacity interfering with host metabolism. This is particularly relevant, as, in addition to the nutrition-dependent formation of microbiota-derived metabolites that can cross the intestinal barrier (e.g. the choline metabolite trimethylamine), cholesterol metabolism, a major pathway that drives atherogenesis, is strongly influenced by the gut microbiota. In recent years, numerous studies have identified the gut microbiota as a relevant source of microbial-associated molecular patterns in plasma. These microbial signatures retain their biologic activity and promote chronic low-grade inflammation, influencing for instance steady-state myelopoiesis and turn-over of myeloid cells but also acting on host metabolic pathways that influence atherogenesis.

Using a germ-free (GF) low-density lipoprotein receptor-deficient (*Ldlr^−/−^*) atherosclerosis mouse model, we tested the microbiota's role in the development of atherosclerotic lesions and arterial thrombosis. Given the complexity of the gut microbial ecosystem, it is evident that such gnotobiotic approaches are invaluable to causally pinpoint the influence of the gut microbiota on plasma cholesterol levels and the size and cellular composition of atherosclerotic lesions at defined diets and standardized feeding regimes. Hence, we fed GF *Ldlr^−/−^* mice with Western diet and compared them to conventionally raised (CONV-R) *Ldlr^−/−^* control mice, which were colonized by a microbiota from birth. Furthermore, these gnotobiotic mouse models are especially interesting to experimentally address whether the absence of microbiota impacts on arterial thrombus growth, the lethal complication arising from the rupture of atherosclerotic lesions.

The basic idea, that colonizing microorganisms might impact disease development of the elderly dates back to Elie Metchnikoff, who in 1908 hypothesized in his book “The Prolongation of Life: Optimistic Studies” that gut-resident microorganisms might be one cause of atherogenesis. At that time of course, Elie Metchnikoff did not have the means to experimentally address the functional role of the microbiota. Nowadays, metagenomics studies on patient cohorts and genetic mouse atherosclerosis models on the depletion of microbiota either by antibiotic treatment protocols or by germ-free housing conditions have identified the commensal microbiota as a modifier of CVD. Furthermore, experimentation with germ-free mouse models has causally linked the gut microbiota and the metabolic capacity of individual gut microbes with host metabolic and signaling pathways that support arterial thrombosis. Therefore, diet-induced dysbiosis, as shown in our recent study (Kiouptsi K, mBio, 2019), could in principle result in an extended phenotype of the gut microbial ecosystem that enhances cardiovascular risk.

Atherogenesis is primarily driven by elevated plasma cholesterol levels and an altered lipoprotein profile. Cholesterol absorption takes place in the upper small intestine. Various experimental and analytical approaches have comprehensively shown that the conversion of cholesterol to the more hydrophilic coprostanol essentially depends on established gut microbial communities **(Figure 1)**. Due to the non-absorbable properties of coprostanol in the small intestine, an inverse relationship exists between serum cholesterol levels and the coprostanol/cholesterol ratio in feces. It is known for many years that the conversion of cholesterol to coprostanol via biohydrogenation is mediated by cholesterol-reducing enzymatic activities of *Eubacterium*, *Bifidobacterium*, *Lactobacillus* and *Peptostreptococcus* strains, promoting cholesterol excretion. Furthermore, in the small intestine, the bile salt hydrolases from different gut bacterial genera deconjugate bile salts and reduce their solubility and reabsorption (**Figure 1**). By studying GF *Ldlr^−/−^* mice relative to their CONV-R *Ldlr^−/−^* counterparts, our study confirmed increased plasma cholesterol levels and elevated very low-density lipoprotein (VLDL), low-density lipoprotein (LDL) and high-density lipoprotein (HDL) levels in this GF *Ldlr^−/−^* hyperlipidemia model when mice were kept on chow diet. However, this difference was abolished when the *Ldlr^−/−^* mice were fed a 0.2% cholesterol-rich diet for 16 weeks. This result was in line with earlier studies on GF apolipoprotein E-deficient (*Apoe^−/−^*) mice that also showed increased plasma cholesterol levels at chow diet conditions, but not at Western diet feeding, when compared to CONV-R controls, demonstrating unequivocally the cholesterol lowering impact of the gut microbiota. Hence, the targeted modulation of gut microbial communities towards communities with enhanced cholesterol biohydrogenation capacity and bile salt hydrolase activity could in the future hold promise for new cholesterol-lowering therapies.

**Figure 1:**
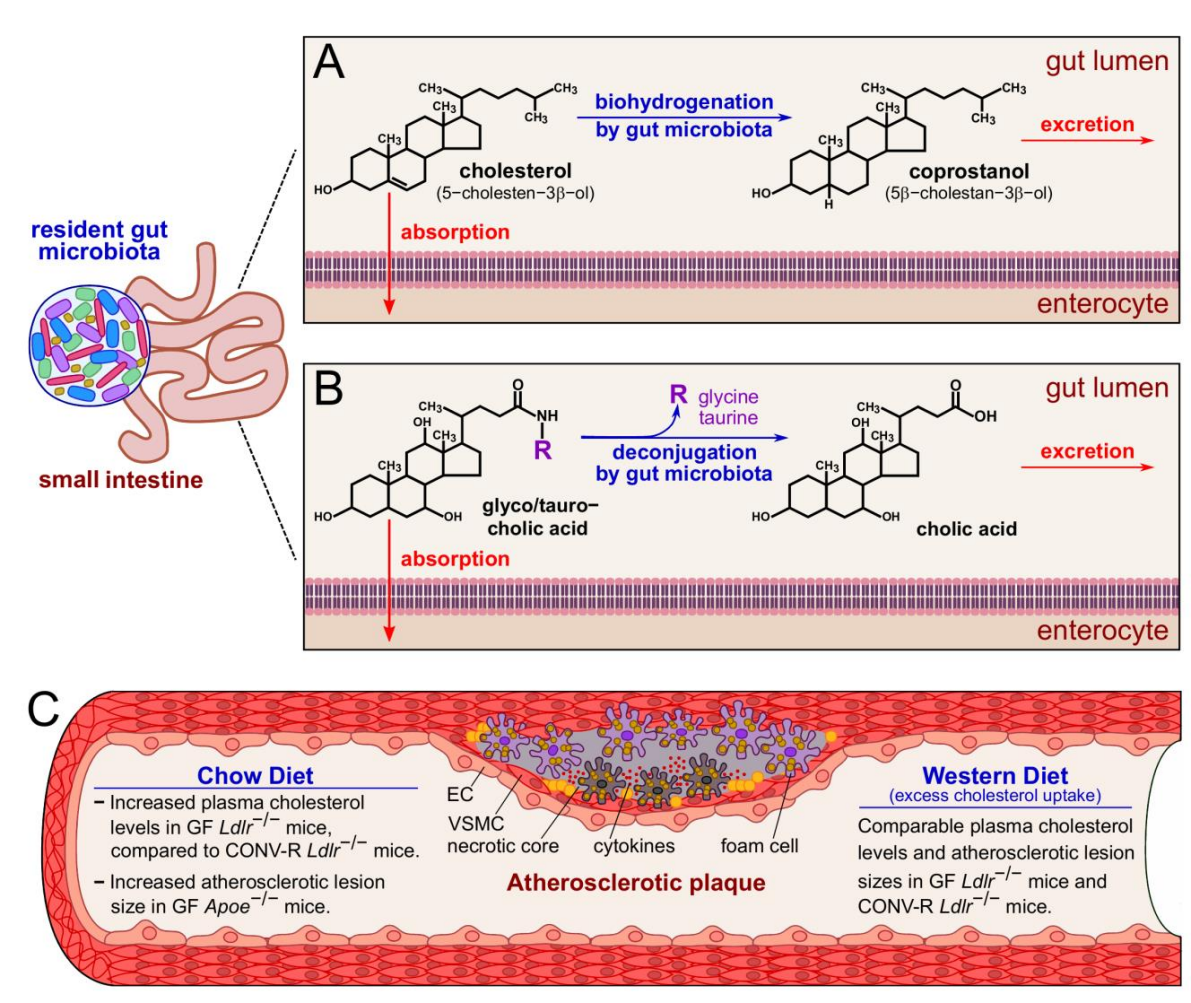
FIGURE 1: Impact of the gut microbiota on cholesterol and bile salt excretion and on and atherosclerotic lesion size under chow diet or Western diet. **(A)** In the gut lumen, the biohydrogenation activity of the gut microbiota converts cholesterol into the more hydrophilic coprostanol. **(B)** The gut microbiota is able to deconjugate both the bile salts glycocholic and taurocholic acids to cholic acid. Coprostanol and cholic acid are readily excreted with the feces. **(C)** In blood vessels, a surplus of circulating cholesterol promotes the formation of atherosclerotic plaques, characterized by an accumulation of smooth muscle cells and macrophages that convert into foam cells. Excess of foam cells leads to apoptosis, necrotic core formation and, eventually, plaque rupture. At chow diet conditions, the gut microbiota prevents cholesterol accumulation, and indeed GF *Ldlr*^−/−^ and GF *Apoe*^−/−^ mice have increased plasma cholesterol levels compared to CONV-R controls. At chow diet conditions, GF *Apoe*^−/−^ mice show increased atherosclerotic lesion size, compared to CONV-R counterparts. On the other hand, excess cholesterol uptake at Western diet conditions counteracts the protective effect of the gut microbiota, and GF and CONV-R *Ldlr*^−/−^ mice show comparable plasma cholesterol levels and atherosclerotic lesion sizes. *Abbreviations:* EC, endothelial cell; VSMC, vascular smooth muscle cell; GF, germ-free; CONV-R, conventionally raised.

As elevated plasma cholesterol levels are a critical determinant for the progression of atherosclerotic lesions, our study on the GF *Ldlr^−/−^* mouse model addressed whether the presence of gut microbiota affects the development of carotid artery lesions. Although we found myeloid blood cell counts significantly reduced in hyperlipidemic GF *Ldlr^−/−^* mice and lower counts of adherent leukocytes to the vessel wall of carotid artery plaques, relative carotid artery plaque areas did not differ between late atherosclerotic GF *Ldlr^−/−^* mice and their CONV-R *Ldlr^−/−^* counterparts. This could be explained by the relatively late time point of our atherothrombosis study (16 weeks of Western diet). Alternatively, the unchanged plasma cholesterol levels and lipoprotein profile of late atherosclerotic GF and CONV-R *Ldlr^−/−^* mice might be due to the relatively high cholesterol content (0.2%) of the Western diet. Future studies should therefore test standardized diets with lower cholesterol content and analyze whether the absence of the gut microbiota affects the time course of atherogenesis in the *Ldlr^−/−^* mouse model. Based on our results, further in-depth analyses of gnotobiotic mouse models on atherosclerotic lesion size and plaque composition are required to pinpoint whether the cholesterol-lowering impact of the gut microbiota or the pro-inflammatory roles of gut-resident commensals can influence the onset and time course of atherogenesis.

As atherosclerotic lesions in the carotid arteries are a frequent cause of stroke, our study put focus on atherothrombosis, the lethal complication of atherosclerosis. In our previous work, we have shown that the gut microbiota promotes carotid artery thrombosis even at chow diet conditions. In addition to the carotid artery ligation model that has revealed reduced platelet deposition to exposed subendothelial matrix in GF relative to CONV-R C57BL/6J mice at chow diet conditions, our recent study demonstrated that GF C57BL/6J mice kept on chow diet also show significantly reduced occlusion times in the ferric chloride injury model of carotid artery thrombosis. At chow diet conditions, pattern recognition of microbiota-derived ligands by hepatic endothelial cells influenced arterial thrombus growth through modulation of hepatic endothelial von Willebrand factor (VWF) synthesis and VWF plasma levels. In contrast, at Western diet conditions, the adhesion-dependent platelet deposition to type III collagen, a major constituent of atherosclerotic lesions, was significantly reduced in GF *Ldlr^−/−^* mice relative to CONV-R *Ldlr^−/−^* mice in standardized flow chamber experiments with anticoagulated whole blood **(Figure 2)**. Interestingly, we observed a diminished exposure of the platelet activation marker phosphatidylserine on the outer leaflet of the platelet membrane of GF *Ldlr^−/−^* mice in this standardized flow chamber model. Thus, our data indicate that at Western diet feeding conditions, the presence of gut microbiota promotes adhesion-dependent platelet activation. This observation is largely coherent with recent studies from other laboratories that have linked the gut microbiota with diet-dependent platelet hyperreactivity and demonstrated that fecal microbiota transplantation potentially can influence procoagulant function.

**Figure 2:**
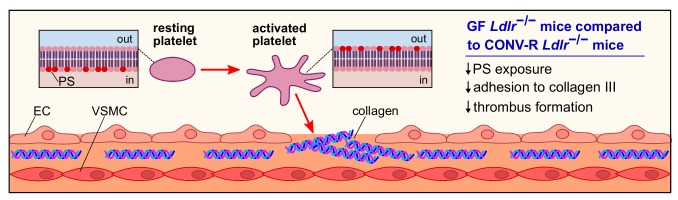
FIGURE 2: Platelet activation and adhesion in GF and CONV-R *Ldlr^−/−^* mice. Platelet activation is accompanied by the flipping and exposure of the procoagulant phosphatidylserine from the inner to the outer leaflet of the membrane. Compared to CONV-R counterparts, GF *Ldlr*^−/−^ mice show reduced phosphatidylserine exposure, yielding in a lower adhesion of platelets to subendothelial type III collagen and impaired thrombus formation. *Abbreviations:* PS, phosphatidylserine; EC, endothelial cell; VSMC, vascular smooth muscle cell.

Collectively, our study argues for a prothrombotic role of the microbiota under hyperlipidemic Western diet conditions that is due to enhanced adhesion-dependent platelet activation on type III collagen in colonized *Ldlr^−/−^* mice and results in a platelet phenotype of increased phosphatidylserine exposure **(Figure 2)**. A number of recent studies have linked the microbiota with metabolic functions that promote platelet reactivity and arterial thrombosis, e.g. by the microbiota's positive impact on the host serotonin biosynthesis pathway or the conversion of choline to trimethylamine. Our study provides an alternative role of the gut microbiota in the modulation of platelet function, as we could show in a standardized flow chamber model that the extent of phosphatidylserine exposure in the platelets of hyperlipidemic *Ldlr^−/−^* mice was dependent on the presence of the commensal microbiota. Since at low cholesterol nutrition the gut microbiota protects the host from high blood cholesterol levels, mechanistic insights on how the metabolic capacity of specific community members affects arterial thrombosis might in the future turn out instrumental to develop new therapeutic interventions based on nutrition, prebiotics or the selective inhibition of metabolic functions of gut microbes to prevent cardiovascular risk.

